# Supplementary material for: Are carbon emissions trading and green financial instruments synergistic? -Comprehensive quantitative research based on content analysis
Source: PLoS One. 2024 Mar 7;19(3):e0298601. doi: 10.1371/journal.pone.0298601 (PMC10919601; doi:10.1371/journal.pone.0298601)
Supplement: S1 Data — (ZIP) [file pone.0298601.s001.zip › Data/地级市Z_F工作报告.zip!/║╙─╧/░▓╤⌠╩╨/└┤╫╘╣1⁄2╓┌║┼í╛┬φ┐╦╩2╛▌═°í┐_2014.docx]

各位代表：

　　现在，我代表市人民政府，向大会报告政府工作，请予审议，并请各位政协委员提出意见。

**一、过去五年工作回顾**

　　2009年以来，面对内外矛盾叠加的严峻形势和转型攻坚的艰巨任务，在省委、省政府和市委的正确领导下，全市上下认真贯彻落实科学发展观，努力稳增长、调结构、促转型、破瓶颈、惠民生，同心协力，务实重干，经济社会发展总体持续、总体提升、总体协调、总体有效。

　　——过去五年，是我市战危机、保增长，努力保持经济平稳较快发展的五年。面对国际金融危机严重冲击，我们不为复杂形势所困，不为严峻挑战所惧，把稳增长摆在更加突出位置，从关键环节和紧迫任务入手，从扩亮点、补短板抓起，分类施策，科学应对，化危为机，先后制定实施了“八个方面十大工程”、“8415推进工程”、“8155投资促进计划”、促进困难行业转型解困专题方案等一系列政策措施，建立完善了联审联批联办、领导干部分包联系重点企业等制度，成立了帮扶重点行业、重点企业工作组，大力开展银企对接、产销对接，综合实力显著增强。2013年全市生产总值1684亿元，年均增长10.6%；人均生产总值3.3万元，是2008年的1.6倍；规模以上工业增加值772亿元，是2008年的1.5倍；全社会固定资产投资1369亿元，年均增长19.3%；社会消费品零售总额533亿元，是2008年的2.2倍；公共财政预算收入92.2亿元、支出216.8亿元，分别是2008年的1.8倍和2.3倍；城镇居民人均可支配收入23019元，农民人均纯收入9670元，分别是2008年的1.7倍和1.9倍。

　　——过去五年，是我市调结构、促转型，不断加快发展方式转变的五年。把加快发展方式转变作为关系全局的紧迫而重大的战略任务，在优化结构中稳增长，在创新驱动中促转型，发展质量和效益明显提高。着力构建科学发展载体。按照“三规合一”、“四集一转”、产城融合的理念，规划建设了9个产业集聚区、20个专业园区，初步形成了钢铁精深加工、装备制造、电子信息、建筑陶瓷、新能源、食品医药等一批特色产业集群，集聚集约发展态势明显增强。2013年产业集聚区固定资产投资和规模以上工业增加值对全市的贡献率分别达到30%、70%以上，成为转型升级的突破口和经济发展的重要增长极。积极推动工业转型升级。深入实施工业振兴工程和嫁接式转型开发，扎实推进传统产业转型和新兴产业培育，优化存量，扩充增量，产业规模层次不断提升。五年累计实施532个打基础、增后劲、创优势的省市重点项目，完成投资1230.6亿元。安钢冷轧薄板、安化PET等一批转型升级项目建成投产。安彩光伏玻璃、大通微电子、光远新材料等一批战略性新兴产业项目快速发展。联合国工业发展组织太阳能光伏研发基地落户我市。14家企业入围河南省百强、百高企业。加快发展现代服务业。大力实施文化旅游发展战略，积极推进“八个园”、“八个一”和十大文化旅游工程建设，着力加快物流快递、信息服务、电子商务、金融保险、房地产等现代服务业发展，有力拉动了经济、繁荣了城市。五年接待国内外游客突破1亿人次，旅游总收入682.2亿元。荣获“国际知名旅游目的地”称号。中国文字博物馆震撼开馆，曹操高陵成功发掘。市会展中心、华强建国酒店、沃尔玛商场、义乌国际商贸城、丹尼斯百货等建成投用。安阳物流港、众品冷链物流等投入运营。9个商务中心区和特色商业区加快推进。我市被批准为国家通用航空产业园区试点。努力提升创新能力。五年新增国家级高新技术企业36家，市级以上研发中心133个，启动实施重大科技专项15个。国家重点实验室和中国驰名商标实现零的突破。连续3次荣获全国科技进步先进市称号，成功创建国家知识产权试点市。科技进步对经济的贡献率达到54%。节能减排成效显著，单位生产总值能耗下降22.6%，环境质量逐步改善。

　　——过去五年，是我市强基础、促增收，扎实推进农业现代化的五年。认真落实强农惠农富农政策，扎实推进高标准粮田“百千万”建设和农业产业化集群培育工程，农业农村发展形势良好。粮食总产达到70.86亿斤，净增5.22亿斤，连续11年增产增收。滑县连续11年荣获全国粮食生产先进县称号。加快农业结构调整。高效农业面积达到110万亩。新建现代农业园区53个、大中型规模养殖场849个。新发展市级以上龙头企业131家。新增农民专业合作社2820家。培育家庭农场738家。加强农业基础设施建设。大中小型水库和农田水利基本建设全面加强。南水北调配套工程主体完工。连续19年夺得省“红旗渠精神杯”。新建大中型沼气池345座。解决了155万人饮水安全问题。完成341个贫困村整村推进任务，25.8万农村贫困人口稳定脱贫。加强生态建设。五年累计营造林142万亩，新增城市绿地317万平方米，森林覆盖率由14.8%提高到21.3%。汤河湿地公园被批准为国家湿地公园。成功创建全国绿化模范城市、全国节水型社会建设示范区。

　　——过去五年，是我市抓引领、促协调，着力统筹城乡发展的五年。突出新型城镇化引领，加快城市组团发展，城市功能不断完善，综合承载能力进一步提升。2013年城镇化率达到43.8%，比2008年提高6.5个百分点。坚持中心城市带动。按照“南扩、东进、西移、北联”的思路，积极推进中心城区和城市组团联动发展。安阳新区、安汤新城加快建设。安阳高新区晋升为国家级高新区。加强城市建设和管理。大力实施城建三年计划和城建三年大变样暨都市区建设，着力打造“净、绿、亮、美、畅”城市环境。旧城、“城中村”改造稳步推进。水电气暖等基础设施不断完善。中华路南北段、京港澳高速公路文峰立交桥等“六桥一互通”竣工。成功创建国家卫生城市和国家园林城市。被评为国家可再生能源建筑应用示范城市、河南省精细化管理优秀城市。完善综合交通体系。安南高速公路、林长高速公路建成通车，晋豫鲁铁路进展顺利。石武高铁安阳段投入运行。豫东北机场选址确定。航空港、陆路港加快建设。“三纵二横一联”国道路网体系和横贯东西、纵穿南北的立体交通格局初步形成。我市被国家确定为地区性综合交通枢纽城市和国家二级物流园区布局城市。促进城乡协调发展。连续12年被评为全省村镇建设先进市。新型农村社区稳妥推进。农村卫生、文化、交通等公共服务明显改善。新建改建农村公路2668公里。19个乡镇入围河南省百强乡镇，水冶镇连续9年保持综合实力第一名。

　　——过去五年，是我市抓改革、促开放，积极增强发展活力的五年。坚持把改革开放作为推动科学发展的根本动力，努力消除体制障碍、提升开放水平，持续积聚发展正能量。加快推进重点领域改革。77户国企改革实现“大头落地”，市属国企产权制度改革基本完成。钢铁、水泥、煤焦化战略重组成效明显。林州重机、顺成煤焦成功上市。安阳海关正式开关。非公有制经济快速发展。交通银行、中信银行、招商银行、浦发银行、郑州银行等落户安阳。安阳银行实现向区域性银行转变。深化行政审批制度改革，取消和下放审批事项72个。财税、教育、科技、水利、集体林权、事业单位、医药卫生等改革加快推进。荣获全国文化体制改革工作先进地区。全面提升开放型经济发展水平。深入开展大招商活动，积极承接产业转移，加强与央企战略合作，成功举办第八届豫商大会，开放招商呈现规模扩大、质量提升、领域拓宽的良好局面。安阳精密制造产业园、万达广场、远大可建、盈德气体等一批龙头型、基地型项目落户我市，中建材、国药集团、华强、林安物流等一批知名企业布局安阳。五年累计引进省外资金形成固定资产投资1507.1亿元，年均增长27.2%；实际利用境外资金12.2亿美元，年均增长32.9%，是我市历史上落户客商最多、招商成效最好的时期。

　　——过去五年，是我市惠民生、促和谐，持续提高人民生活水平的五年。不断加大民生投入，努力使改革发展成果最大程度惠及广大人民群众。全市财政民生支出五年累计617.1亿元，占财政支出的71.9%。加强就业工作。五年城镇新增就业47.3万人，再就业超额完成目标。农村劳动力转移就业新增20万人、总量达到178万人。提升社会保障水平。覆盖城乡的社会保障体系初步建立。城镇居民医保、城乡居民养老保险制度实现全覆盖。新农合参合率达到99.1%。企业退休人员养老金标准连年提高。城乡居民医保补助标准五年提高3.5倍。城乡低保、农村“五保”等社保对象待遇不断提高。多层次住房保障体系初步形成，五年开工建设保障性住房6.7万套，竣工5.1万套。全面发展社会事业。义务教育均衡发展，学前教育三年行动计划圆满完成，城乡免费义务教育全面实施，职教园区初具规模，“三本三专”建设目标圆满完成。全市公共文化服务单位全部免费开放，实现了县县有图书馆、文化馆，乡乡有文化站，村村通广播电视、有农家书屋，覆盖城乡的公共文化服务体系初步建立。基层医疗卫生体系不断完善，卫生机构病床新增3941张，12类基本公共卫生服务免费向城乡居民提供。全民健身运动广泛开展，竞技体育取得新成绩。市场体系建设扎实推进。人事人才工作得到加强。成功创建全国人口和计划生育综合改革示范市、全国人民防空先进城市。加强社会治理。食品药品监管全面加强。全力推进非法集资案件处置。平安安阳建设扎实推进，社会治安、信访和安全生产形势持续好转，社会大局总体稳定。

　　精神文明和民主法治持续加强。坚持依法行政，全面推进法治政府建设。自觉接受人大法律监督、工作监督和政协民主监督，认真办理人大代表议案、建议和政协提案。认真落实中央八项规定精神，深入推进反腐倡廉建设，切实加强行政效能监察。坚持军民融合式发展，军政军民团结取得新成效，第五次被命名为全国“双拥”模范城。老区建设不断加强。依法保护妇女儿童合法权益。社会福利、慈善事业和老龄、红十字、残疾人、关心下一代工作健康发展。统计、审计、史志、档案、气象、供销、地震、邮政、发展研究、外事侨务、民族宗教等工作取得新成绩。

　　各位代表！

　　刚刚过去的2013年，是我市科学应对危机、经济稳定回升的一年，也是取得显著效果的一年。一年来，面对世界经济复苏艰难、国内经济下行压力加大、多重矛盾交织的复杂形势，我们坚持稳中求进、持续提升工作总基调，统筹稳增长、调结构、促改革，制定出台了支持工业发展的39条意见、促进房地产和建筑业健康发展意见等一系列针对性强的政策措施，扎实推进产业转型升级、新型城镇化、项目建设、开放招商等事关全局的重点工作，经济社会发展稳中有为、稳中提质、稳中有进。全市生产总值增长8.5%，公共财政预算收入增长10.3%，全社会固定资产投资增长22.2%，社会消费品零售总额增长13.6%，城镇居民可支配收入、农民人均纯收入分别增长9.4%和12.2%。2013年各项目标任务的圆满完成，标志着我市在全面建成小康社会的征程上迈出了坚实步伐，踏上了新的起点。

　　各位代表！

　　过去五年，困难比预料的多，结果比预想的好，成绩来之不易。这是市委正确领导的结果，是市人大、市政协大力支持的结果，是全市人民齐心协力、团结拼搏的结果。在此，我代表市人民政府，向奋斗在全市各条战线上的广大工人、农民、干部、知识分子，向解放军指战员、武警官兵、政法干警以及各界人士，致以崇高的敬意！向所有关心支持我市发展的港澳台同胞、海外侨胞、国际友人，表示诚挚的感谢！

　　总结成绩的同时，我们也清醒地认识到，全市经济社会发展还面临很多困难和挑战，政府工作还存在不少问题和差距。一是长期形成的粗放型增长方式没有根本扭转，产业结构不优、质量效益不高、创新能力偏弱、产品附加值低等深层次矛盾依然突出，转型升级任务艰巨。二是部分行业和企业生产经营仍然困难，经济回升向好基础还不稳固，发展活力和动力仍然不足。三是农业增产农民增收难度加大，城区经济发展滞后，城区基础设施和公共服务设施还不够完善，城乡统筹发展有待进一步加快。四是民生投入欠账较多，涉及群众切身利益的就业、就学、就医、住房、养老、食品药品安全等问题尚未得到很好解决，非法集资对金融秩序和社会稳定的影响仍然较大。五是政府职能转变不到位，作风不实、服务不优、用权不公、为政不廉、效能不高等问题仍然存在；一些政府工作人员能力素质与新形势新任务要求不相适应，一些部门“四风”问题仍不同程度存在。对此，我们一定高度重视，认真解决，决不辜负人民群众的期望。

**二、今后五年总体要求和奋斗目标**

　　未来五年，是我市深入贯彻落实党的十八大和十八届三中全会精神、全面建成小康社会的关键五年，也是爬坡过坎、攻坚转型的重要五年，更是励精图治、大有作为的五年。面对加快发展、加速转型、改善民生等多重任务，我们既要保持较快的增长速度，努力缩小与先进地区的差距，又要真正转变发展立足点，切实提高发展的质量和效益。尽管挑战前所未有，但机遇大于挑战。只要我们坚定信心、抢抓机遇、开拓进取、科学发展，就一定能够实现经济社会发展的新跨越，就一定能够与全国全省同步全面建成小康社会！

　　今后五年政府工作的总体要求是：全面贯彻落实党的十八大和十八届二中、三中全会精神，紧紧围绕建设中原经济区区域性中心强市目标，突出“加快产业转型升级”发展主线，更加注重优化经济结构，更加注重全面深化改革，更加注重持续扩大开放，更加注重统筹城乡发展，更加注重保障和改善民生，转中求进，改革创新，切实提高经济发展质量和效益，努力打造安阳经济升级版，促进经济持续健康发展、社会和谐稳定。

　　今后五年的奋斗目标是：

　　——着力推动科学发展，实现经济实力新提升。经济结构调整和发展方式转变取得积极进展，经济增长质量和效益显著提高。主要经济指标高于全省平均水平，2018年全市生产总值突破2500亿元，人均生产总值突破5万元，公共财政预算收入150亿元，城市综合实力明显提升。

　　——着力加快产业升级，实现转型发展新突破。新型工业城市初步建立，工业增加值年均增长11%以上，战略性新兴产业增加值占生产总值的比重在15%左右。文化旅游更加繁荣，文化软实力显著增强。新型农业现代化迈上新台阶，高效农业、现代畜牧业快速发展。信息化水平显著提高，科技进步对经济增长的贡献率明显提高。

　　——着力推进新型城镇化，实现城乡面貌新变化。新型城镇化加速推进，现代城镇体系基本健全。城镇基础设施更加完善，基本公共服务实现常住人口全覆盖，城乡发展一体化格局初步形成。2018年中心城区面积达到138平方公里，人口达到138万人，全市城镇化率在54%以上。

　　——着力加强生态建设，实现生态环境新改善。积极推进绿色安阳建设，发展循环经济，加强环境保护，基本形成绿色低碳发展模式。2018年全市森林覆盖率达25%，节能减排成效显著，生态质量明显提高。

　　——着力深化改革开放，实现发展活力新增强。现代市场体系基本完善，各类市场主体更具活力，市场配置资源的决定性作用更为突出。要素保障长效机制基本健全，土地节约集约利用机制更加完善。政府公共服务和依法行政能力显著提高，有利于科学发展的体制机制基本确立。开放领域更宽、层次更高，开放型经济显著增强。

　　——着力保障和改善民生，实现人民生活新提高。努力实现居民收入增长与经济发展同步、劳动报酬增长和劳动生产率提高同步，收入分配更趋合理。基本公共服务水平和均等化程度全面提高，人人享有基本就业创业机会、基本社会保障和基本医疗卫生服务。人民精神文化生活更加丰富，社会更加和谐稳定。

　　各位代表！

　　站在新的历史起点上，我们深知，人民群众对安阳未来充满了期盼。期盼着经济快速增长，民生日益改善，早日实现富民强市；期盼着城市功能日臻完善，更加宜居宜业宜商；期盼着教育、医疗、文化、商业等公共服务不断完善，改革发展的成果更多惠及全市人民；期盼着政府职能加快转变，服务更高效，办事更方便，发展环境更宽松。这些期盼，既是广大人民群众对美好生活的向往，也是政府工作的目标和重点，我们将为之不懈努力。

　　我们坚信，经过五年或更长时间的奋斗，一个经济日益发达、文化日益繁荣、社会日益和谐、生态日益文明的安阳，一个更有实力、更具活力、更富魅力的安阳，必将更加精彩地展现在世人面前。

**三、2014年工作安排**

　　今年是全面深化改革的第一年，是完成“十二五”规划的关键一年，也是新一届政府的开局之年。总的来看，我市面临的发展环境依然错综复杂。世界经济复苏存在不稳定、不确定因素，外部环境依然偏紧。我国经济进入增长速度换挡期、结构调整阵痛期，改革进入深水区和攻坚期，经济发展稳中有忧、稳中有险。区域竞争更加激烈，稍有懈怠就会陷于被动。特别是我市正处于爬坡过坎、攻坚转型的关键时期，传统优势有所减弱，新的接续支撑力量尚未形成，深层次矛盾没有根本解决，发展的任务十分繁重。但是，也要看到我市发展仍然具有诸多机遇和有利条件。我国经济长期向好的基本面没有改变，全面深化改革将释放新的动力和活力。沿海地区产业、资金、技术加速向中西部地区转移，我市将迎来人才、技术、政策、资金等要素聚集高峰期。同时，我市正处于工业化、城镇化快速发展阶段，基础设施、发展载体、人力资源等支撑能力不断增强，综合竞争优势日益彰显，发展前景看好。因此，我们一定要辩证看待形势，既要坚定信心、抓住机遇，充分利用有利条件，又要保持清醒、开拓进取，积极应对复杂局面，以转变经济发展方式的主动、调整经济结构的主动、改革开放的主动，赢得发展上的主动、竞争中的主动。

　　今年经济社会发展主要目标是：生产总值增长9%左右；公共财政预算收入增长10%；全社会固定资产投资增长22%；社会消费品零售总额增长14%左右；外贸出口增长10%左右；城镇居民人均可支配收入实际增长9%左右，农民人均纯收入实际增长9%以上，力争与经济增长同步；城镇新增就业6.5万人以上；人口自然增长率控制在6.4‰以内；居民消费价格涨幅控制在3.5%以内；节能减排力争完成省下达目标任务。

　　实现上述目标，完成今年各项任务，必须坚持“总要求”，转中求进，改革创新；强化“三动力”，通过深化改革、扩大开放、创新发展为加快发展提供强大动力；突出“五着力”，着力扩大需求稳增长，着力优化结构促转型，着力改革创新增后劲，着力创造优势强支撑，着力改善民生促和谐，促进经济持续健康发展、社会和谐稳定。

　　今年着重抓好以下九个方面的工作：

　　(一)突出产业转型，加快建设新型工业城市。坚持优存量与扩增量并举，不断增强产业竞争力和科技创新能力，打造功能完善、结构合理、相互配套的现代产业体系。推动传统产业转型升级。按照有保有压、重点突破、提升档次的原则，综合运用延伸链条、技术改造、兼并重组等手段，对传统支柱产业进行脱胎换骨式的改造提升。依托安钢和沙钢永兴集团，加快发展钢材精深加工，打造全省精品钢生产基地。依托河南能源集团安化公司和河南中安煤化集团，大力发展环保节能、清洁、循环型煤化工产品，打造全省知名新型煤焦化产业基地。依托安阳精密制造产业园，发展整机装备和高端配套产品，打造研发水平高、技术先进、系统配套能力强的先进装备制造业基地。依托凤竹纺织集团，加快规划建设中原高新技术产业开发区，打造重要的纺织服装基地。加快新兴产业培育。重点发展新能源、生物医药、电子信息、新材料、航空制造等高成长性、战略性新兴产业。以安彩高科等为重点，大力发展平板显示器、电器元件、通讯产品等电子信息产业，促进电子信息产业整体做大、局部做强、集群发展。以中农颖泰、东泰制药等为龙头，打造新医药研发生产基地。以林州光远等为带动，打造新型材料研发生产基地。发挥安阳航空产业优势，打造轻型飞机、航模等通用航空产业基地。推进发展载体建设。按照总体做强、集群做大、主业突出、产业配套的思路，坚持“四集一转”，促进产业集聚区提质发展。加强载体间的联系，引导同一地域内的工业、服务业和农业发展载体融合发展，不同层级的载体互动发展，相邻地区的载体联动发展，新老载体耦合发展。重点在纺织服装、新型陶瓷、食品医药、精密制造等方面打造百亿级特色产业集群。力争全年完成投资480亿元，规模以上工业主营业务收入1400亿元。强化创新驱动。加强自主创新体系建设，突出高新技术产业发展，新建一批重点实验室、工程技术研究中心、科技企业孵化器。加快推进科技成果转化，促进产学研相结合，鼓励相关企业、高等院校和科研机构建立产业技术创新战略联盟，组织实施一批重大科技专项。实施质量强市战略，争创国家知识产权示范城市。加强信息化与新型工业化、新型城镇化、农业现代化融合发展，加快建设无线城市、智慧安阳。

　　(二)强化引领带动，加快推进新型城镇化。全面落实科学推进新型城镇化指导意见，按照以人为本、四化同步、优化布局、生态文明、传承文化、产城互动、科学有序的原则，积极稳妥推进新型城镇化。推进中心城区和城市组团发展。按照“1+4+1”大市区发展布局，推动中心城区和城市组团完善功能配套、合理布局产业，实现高效连接、联动协调发展。加快安阳城乡一体化示范区基础设施建设和精密制造、电子信息、现代物流等产业发展，打造城区经济增长极。加快安汤新城羑河综合治理开发和生态廊道工程建设，积极发展金融、商贸、高科技产业，增强南部高新技术产业辐射带动能力。加快安北新区规划建设，打造北部历史文化风光休闲旅游带。加快新西区规划建设，推进西部新型工业化和循环经济发展。支持内黄新型陶瓷、汤阴食品医药、水冶钢铁加工、柏庄针织服装等产业发展，支持林州市发展成为中等城市。完善提升老城区功能。加强城市精细化管理，继续开展城市环境综合整治。加快推进旧城和“城中村”、棚户区改造。完善公共交通、生态环保、水电气暖等基础设施和文化、卫生等公共服务设施。开工建设东工路南段，打通太行路、钢城路、朝霞路等断头路。加快推进洹北污水处理厂、第八水厂建设。积极构建综合交通体系。大力实施大交通体系建设三年攻坚计划，全力构建铁路、公路、航空、港口联动发展的现代化交通、物流、商贸发展新格局。加快推进晋豫鲁铁路建设，确保年内具备通车条件。加快推进鹤辉高速林州段工程建设，开工建设西北绕城高速公路，积极做好林桐高速前期工作。加快推进东北环城、西南环城快速路建设，解决市区东北部、西南部交通压力。加快推进安水、安内快速路等干线公路建设。完成安邯、安鹤快速通道建设。加快推进安阳机场和安阳航空港、陆路港建设。科学有序推进以人为核心的新型城镇化。深化户籍制度改革，推动“城中村”、城郊村和产业集聚区内村庄成建制转为城镇户口，有序推进农业转移人口市民化。增强城镇产业支撑能力，更好地解决进城农民就业问题，着力促进城镇基本公共服务常住人口全覆盖。

　　(三)围绕提质增效，加快发展现代农业。大力推进现代农业示范市建设，强化政策支持，培育新型农业经营体系，提高农业现代化水平。继续实施高标准粮田“百千万”建设工程。新建高标准粮田30万亩，粮食种植面积稳定在560万亩，力争总产达到215万吨。继续实施农业产业化集群培育工程。重点抓好5个省级农业产业化集群和一批高成长性产业化集群发展，新发展市级以上重点龙头企业10家，力争农业产业化集群总产值突破160亿元。积极推进现代农业园区建设工程。大力发展高效农业和都市生态农业，加快农产品精深加工园区建设，突出抓好汤河现代农业主题公园、林州红旗渠、内黄新区等6个现代农业综合示范区建设，建设提升10个主导产业示范区和20个特色农业精品园。着力推进农业经营体系创新工程。培育家庭农场、农民合作社、农业企业等新型农业经营主体。有序推动土地承包经营权向新型农业经营主体流转，鼓励引导工商资本到农村发展适合企业化经营的现代种植业。力争新增土地流转面积10万亩。加快推进农业生态保护工程。大力发展资源节约型农业，完善农产品质量安全检测体系和农业服务体系。加快水库除险加固及农田水利基本建设，完成南水北调配套工程。加快“美丽乡村”建设。按照产业、村庄、土地、生态环境和公共设施“五规合一”要求，积极稳妥推进新农村建设。加强农村水电路气等基础设施建设，解决20万人饮水安全问题。新建改建农村公路200公里。新增沼气用户8000户，新建大中型沼气工程30座。完成3万贫困人口脱贫。

　　(四)着力做大服务业，加快繁荣第三产业。把加快服务业发展作为产业转型升级的有效途径，通过改革推动、项目带动和政策引领，促进服务业发展提速、比重提高、水平提升。大力发展文化旅游业。围绕建设国际旅游城，积极推进殷墟国家考古遗址公园、红旗渠太行大峡谷文化旅游产业园、周易文化产业园等十大文化旅游产业园区建设。加快推进安阳·中国历史文化产业博览园建设。做好红旗渠和太行大峡谷捆绑申报国家5A级景区工作。组织开展好第六届安阳航空运动文化旅游节、“韩国万人游安阳”等活动。大力发展低空经济，着力打造航空运动之都。加快发展高成长性服务业。围绕提升现代物流、信息服务、金融等高成长性服务业，鼓励支持一批物流园区、城市配送中心、软件园区、电商产业园、金融集聚区建设。加快推进安阳物流港、安阳世贸中心等项目建设。积极培育新兴服务业。支持科技研发、教育培训、商务服务、健康服务、家庭服务等新兴服务业发展，加快建设专业化第三方研发机构、高端服务业集群、知名会展品牌，培育服务业发展生力军。改造提升传统服务业。加快商务中心区和特色商业区规划建设。积极引入现代管理理念、新型商业模式和信息技术，改造提升传统支柱服务业。促进房地产业健康发展。

　　(五)实施项目带动，加快增强发展后劲。项目建设是扩投资、稳增长、促转型的重要手段。必须坚持项目建设不动摇，优化项目结构，提升项目质量，为长远发展增添后劲。深入实施项目带动战略。谋划实施247个亿元以上重大项目，力争全年完成投资574亿元。抓好工业设计之都、台湾迎辉科技、豫科隆盛空冷设备等战略性新兴产业项目建设。加快安彩高端玻璃、龙宇新材料等工业项目建设。推进希尔顿城市综合体、五洲国际工业博览城、空港新城航空大厦等城建项目建设。做好路港国际综合物流园、泰祥汽博物流产业园、中华梦幻谷等服务业项目建设。积极扩大投资。围绕省三大战略规划和《全国老工业基地调整改造规划》，在铁路、信息消费、城市基础设施、节能环保、保障性安居工程等国家重点支持的领域，谋划实施一批符合产业政策、带动能力强的大项目好项目。强力推进200个省、市重点项目建设。优化项目建设环境。进一步完善重大项目领导干部分包、定期通报和联审联批联办等制度，健全项目考核奖惩长效机制，研究建立重大项目异地利益分享机制。组织开展全市重点项目观摩点评活动。力争新开工项目、竣工项目和年度投资目标100%完成。

　　(六)推进开放招商，加快提升开放型经济水平。充分发挥开放招商“一举求多效”的综合带动作用，以开放促改革、促转型、促发展。拓宽开放领域。抓住国家放宽外商投资市场准入机遇，推进投资管理、金融制度、服务业开放和贸易监管制度创新，加快服务业、农业、基础设施和社会事业等领域开放，引进一批具有战略意义的大项目。力争年内引进省外资金突破500亿元。创新招商方式。紧盯沿海发达地区和央企、国内外500强、行业知名企业，大力开展区域招商、以商招商、中介招商和“一对一”叩门招商，提升招商专业化水平，实现产业链式转移和集群式引进。提升招商质量。注重企业和项目发展的可持续性，积极引进竞争力强、成长性好、带动作用明显的项目，引进先进技术、管理、人才及研发机构。重点抓好林安物流、国家工业设计研究院、国药物流等项目落实。优化投资环境，切实保护投资者合法权益。拓展外需市场。支持企业“走出去”，积极组织参加广交会、高交会等境内外大型经贸活动。扩大先进技术设备、关键零部件、能源原材料进口，培育壮大出口十强、进口十强和100家有出口实绩的企业。

　　(七)坚持改革创新，加快培育发展新优势。按照中央和省委的部署，以经济体制改革为重点，加快一批看得准、有条件、有权限的改革，努力在重点领域、关键环节取得实质进展。深化国有企业改革。继续推进国企改革，基本完成市属国有企业改革扫尾任务，妥善解决企业改革历史遗留问题，鼓励非公有制企业参与国有企业改制重组。支持非公有制经济健康发展，鼓励有条件的私营企业建立现代企业制度，吸引民间资本进入交通、能源、市政设施、社区服务等领域。扎实推进财税体制改革。深化政府和部门预决算公开，改进年度预算控制方式。清理规范税收优惠政策，全面落实国家“营改增”、资源税从价计征等改革政策。加强预算绩效管理，从严控制“三公经费”等一般性支出，盘活财政存量资金。加强政府债务管理，有效防范和化解财政风险。推动投融资平台整合功能，提升投融资能力、资产管理能力和风险防范能力。积极促进金融改革。大力引进各类金融机构，全面建立风险补偿机制，优化小微企业融资环境，防范和化解金融风险。积极探索乡镇金融改革，盘活农民资产，搞活农村金融。力争年内培育3家上市企业。同时，认真推进文化、医药卫生、土地管理体制、资源性产品价格等改革。

　　(八)加强生态环境保护，加快建设美丽安阳。牢固树立保护生态环境就是保护生产力、改善生态环境就是提升竞争力的理念，更加自觉地推进绿色发展、循环发展、低碳发展，增强可持续发展能力。切实改善环境质量。实施蓝天工程，推动重点行业、重点部位大气污染防治，加大市区扬尘、机动车尾气污染防治力度，有效遏制雾霾污染。实施碧水工程，加强污水处理厂配套设施建设和运行管理，加快安阳河、洪河和茶店河治理，推进全市河渠坑塘环境整治，有效改善水环境质量。加强农村面源污染治理，推进农村环境综合整治，改善农村生态环境。加强资源能源节约。以节能降耗为重点，提高资源节约集约利用水平。大力发展循环经济，加快推进安西循环经济区建设。严格环保准入和污染物排放标准，加强工程减排、结构减排、管理减排。加强生态体系建设。落实生态市建设规划，实施林业生态市建设提升工程，加强生态体系、生态廊道网络建设。抓好以高速公路为重点的通道绿化，营造林20万亩，通道绿化工程造林1.9万亩。积极推进全省水生态文明试点城市建设，加快完善城乡生态水系。

　　(九)切实保障和改善民生，加快建设平安安阳。政府工作的根本目的，是让全体人民过上好日子。要继续抓好“十项民生工程”，使发展成果更多更公平惠及全市人民。推动实现高质量就业。实施更加积极的就业政策，优化就业创业环境，以创新引领创业，以创业带动就业，使劳动者生活更加体面、更有尊严。重点做好高校毕业生、城镇困难人员、退役军人等就业。实现再就业2.4万人，其中，困难群体就业0.8万人。新增农村劳动力转移就业133万人。确保“零就业”家庭至少有一人就业。努力增加居民收入。完善机关事业单位工资和津补贴制度，推行企业工资集体协商，适时调整最低工资标准。增加城乡居民财产性收入，不断提高消费能力。加强社会保障体系建设。坚持保基本、兜底线、促公平、可持续，继续扩大社会保障覆盖范围。提高企业退休人员养老金。推进城镇企业职工基本养老保险与城乡居民社会养老保险衔接。城镇居民医保和新农合人均财政补助标准提高到320元。健全社会救助制度，落实好社会救助和保障标准与物价上涨联动挂钩机制。推进廉租房、公租房并轨运行，开工建设保障性住房16429套，竣工12882套。完善减灾救灾、社会救助、社会福利制度，大力发展老龄、残疾人、妇女儿童、红十字、慈善事业，让每一个身处困境者都能得到社会关爱和温暖。加快发展各项社会事业。继续抓好学前教育，促进义务教育均衡发展，推动普通高中多样化发展，支持高等教育内涵式发展，努力办好人民满意的教育。深化医药卫生体制改革，巩固完善基本药物制度。加强基层医疗卫生服务体系建设，促进优质医疗资源纵向下沉。积极发展中医药事业。加强公共文化设施建设，扎实推进文化惠民工程，建设数字多媒体应急信息发布系统。完善社会养老服务体系，积极发展养老事业。调整完善生育政策，促进人口长期均衡发展。实施老年体育“长青工程”，广泛开展全民健身运动，提高竞技体育水平。加强和创新社会治理。完善社会稳定风险评估机制，强化应急管理体系建设。强力推进非法集资专项整治。全面落实安全生产责任制，坚决遏制重特大安全事故发生。加强食品药品监管，用最严格的监管、最严厉的处罚、最严肃的问责，切实保障“舌尖上的安全”。强化信访工作和社会管理综合治理，有效排查化解社会矛盾，维护社会大局稳定。

　　深入开展中国特色社会主义和中国梦宣传教育，大力培育和践行社会主义核心价值观。深入推进公民道德建设，提升全民文明素养。继续加强国防教育和国防后备力量建设，支持驻安解放军、武警部队、民兵和人民防空建设。深入开展“双拥”共建，促进军民融合式发展。加强老区建设。支持工会、共青团、妇联等人民团体发挥桥梁纽带作用。认真做好关心下一代工作。继续做好统计、审计、档案、史志、气象、地震、邮政、发展研究、外事侨务、民族宗教等工作。

**四、努力推进政府自身建设**

　　加快安阳转型发展、和谐发展、科学发展，人民寄予厚望，政府责任重大。要把转变职能和作风、强化依法行政、提升能力素质作为政府自身建设的关键，深入推进“两转两提”，不断提高政府工作水平。

　　(一)转变政府职能。强化政府公共服务、市场监管、社会管理、环境保护等职责，推动政府职能向创造良好发展环境、提供优质服务、维护社会公平正义转变。继续清理和精简行政审批事项，该放的事项坚决放开放到位，该管的事情切实管住管到位。推进工商注册制度便利化，由先证后照改为先照后证。加强政府绩效管理，修改完善政府责任目标考核体系，引导转型升级和科学发展。深化政府机构改革，加强事业单位分类改革，严格机构编制管理。

　　(二)推进依法行政。严格按照法定权限和程序行使权力、履行职责，推进政府工作法治化。自觉接受人大及其常委会的法律监督、工作监督和政协的民主监督。广泛听取各民主党派、工商联、无党派人士的意见和建议。加强行政监察和审计监督，虚心接受社会监督和舆论监督。坚持依法科学民主决策，完善重大决策合法性审查和风险评估机制。深入推行服务型行政执法，强化行政执法责任和过错追究，积极推进行政复议委员会试点工作。深化政务公开，让人民监督权力，让权力在阳光下运行！

　　(三)改进工作作风。坚持人民至上，始终把人民满意作为第一标准，扎实开展党的群众路线教育实践活动。深入落实中央八项规定精神，持续解决“四风”突出问题，健全改进作风常态化制度，提升政府执行力和公信力。加强政务诚信建设，以政务诚信带动商务诚信和社会诚信。深化学习型机关建设，强化教育培训，增强公务员队伍的公仆意识、责任意识和能力素质。大力倡导务实重干，努力打造一支信念坚定、为民服务、勤政务实、勇于担当、清正廉洁的公务员队伍。

　　(四)坚持从严治政。加强廉政建设，完善惩防体系，强化纠风和专项治理，坚决查处权力寻租、权钱交易和损害群众利益的行为，以反腐倡廉实效保障发展、取信于民。坚持从严从俭，健全厉行节约反对浪费制度体系，严格控制一般性支出，确保“三公”经费只减不增，禁止新建楼堂馆所，在政府过紧日子中让人民过上好日子！

　　各位代表！

　　百舸争流，千帆竞进。让我们紧密团结在以习近平同志为总书记的党中央周围，在市委的坚强领导下，以更加开阔的视野、更加昂扬的斗志、更加扎实的作风，解放思想、开拓创新、奋力拼搏，为加快建设中原经济区区域性中心强市、为安阳人民生活得更加幸福而努力奋斗！
